# Pancreatic Exocrine Insufficiency after Bariatric Surgery

**DOI:** 10.3390/nu9111241

**Published:** 2017-11-13

**Authors:** Miroslav Vujasinovic, Roberto Valente, Anders Thorell, Wiktor Rutkowski, Stephan L. Haas, Urban Arnelo, Lena Martin, J.-Matthias Löhr

**Affiliations:** 1 Center for Digestive Diseases, Karolinska University Hospital, SE-141 86 Stockholm, Sweden; miroslav.vujasinovic@sll.se (M.V.); roberto.valente@karolinska.se (R.V.); wikru@hotmail.com (W.R.); stephan.haas@karolinska.se (S.L.H.); urban.arnelo@sll.se (U.A.); lena.martin@ki.se (L.M.); 2 Digestive and Liver Disease UnitSant' Andrea Hospital, Sapienza University of Rome, 116 91 Rome, Italy; 3 Department of Clinical Science, Danderyd Hospital, Karolinska Institutet and Department of Surgery, Ersta Hospital, SE-116 91 Stockholm, Sweden; anders.thorell@erstadiakoni.se

**Keywords:** pancreas, exocrine, insufficiency, obesity, bariatric surgery

## Abstract

Morbid obesity is a lifelong disease, and all patients require complementary follow-up including nutritional surveillance by a multidisciplinary team after bariatric procedures. Pancreatic exocrine insufficiency (PEI) refers to an insufficient secretion of pancreatic enzymes and/or sodium bicarbonate. PEI is a known multifactorial complication after upper gastrointestinal surgery, and might constitute an important clinical problem due to the large number of bariatric surgical procedures in the world. Symptoms of PEI often overlap with sequelae of gastric bypass, making the diagnosis difficult. Steatorrhea, weight loss, maldigestion and malabsorption are pathognomonic for both clinical conditions. Altered anatomy after bypass surgery can make the diagnostic process even more difficult. Fecal elastase-1 (FE1) is a useful diagnostic test. PEI should be considered in all patients after bariatric surgery with prolonged gastrointestinal complaints that are suggestive of maldigestion and/or malabsorption. Appropriate pancreatic enzyme replacement therapy should be part of the treatment algorithm in patients with confirmed PEI or symptoms suggestive of this complication.

## 1. Introduction

Obesity is the most prevalent metabolic disease worldwide, and represents a global epidemic in both developed and developing countries [1,2]. Morbid obesity is a lifelong disease, and all patients undergoing bariatric procedures require complementary follow-up, including nutritional surveillance by a multidisciplinary team.

Pancreatic secretions play an essential role in digestion, and are controlled by a host of neuronal and hormonal signaling pathways which modulate not only secretion, but also the cellular integrity of the gland [3]. Neural regulation of this secretion involves the enteric nervous system in the gut and the central nervous system. Autonomic nerves of the pancreas form a separate “pancreatic brain”, which is a part of the enteric nervous system and is responsible for the regulation of the pancreatic secretory function and pancreatic blood flow [4,5]. Efferent parasympathetic system pathways, consisting of central dorsal motor nucleus of the vagus and peripheral pancreatic neurons, stimulate exocrine secretion. Sympathetic innervation of the exocrine pancreas is primarily indirect, and inhibits secretion by decreasing blood flow and inhibiting transmission in the pancreatic ganglia [5]. Factors controlling appetite and energy intake also influence the overall digestive function of the gastrointestinal system involving the pancreas.

Cholecystokinin (CCK) is known to induce pancreatic exocrine secretion by the activation of CCK1 receptor-mediated signaling pathways, but other hormones also play an important role in pancreatic function, including ghrelin, leptin, melanocortin, obestatin, apelin, orexin-A and B and glucagon-like peptide-1 (GLP-1)—Table 1 [3,4,6,7]. GLP-1 and its metabolites have important extrapancreatic effects, particularly with regard to the cardiovascular system and insulinomimetic effects with respect to glucose homeostasis. These effects may be particularly important in obesity [6]. Leptin was the first appetite regulator with an established role in controlling the exocrine pancreas function [8]. Ghrelin is produced predominantly by the gastric mucosa, and significant elevations were observed in plasma just before meals, suggesting its role as a hunger signal [7]. Obestatin is a recently identified acid peptide that appears to have opposite actions to ghrelin on the regulation of food intake, the emptying of the stomach, and body weight in experimental animals [9]. Apelin is secreted by adipose tissue, and its production is increased in obesity. In the gastric mucosa, apelin is involved in gastric cell proliferation, and in controlling exocrine and endocrine functions [10,11,12].

The presence of nutritional deficiencies in obese individuals before the operation may seem paradoxical in light of excess caloric intake, but recently published literature has documented that several micronutrient deficiencies may be present in obese individuals, including adults undergoing preoperative evaluations for bariatric surgery [13]. The causes of nutritional deficiencies in obese individuals has not been fully elucidated, but can in large part be explained by the intake of sweet and high caloric food with poor nutritional quality [14].

## 2. Bariatric Surgery

Bariatric surgery is one of the fastest-growing operative procedures performed worldwide, with more than 500,000 procedures per year [17,18].

Surgery is the most effective treatment for morbid obesity in terms of sustained weight loss, improvement of comorbidities and quality of life, and in terms of the long-term reduction of overall mortality [1,19,20,21,22,23,24].

**Figure 1:**
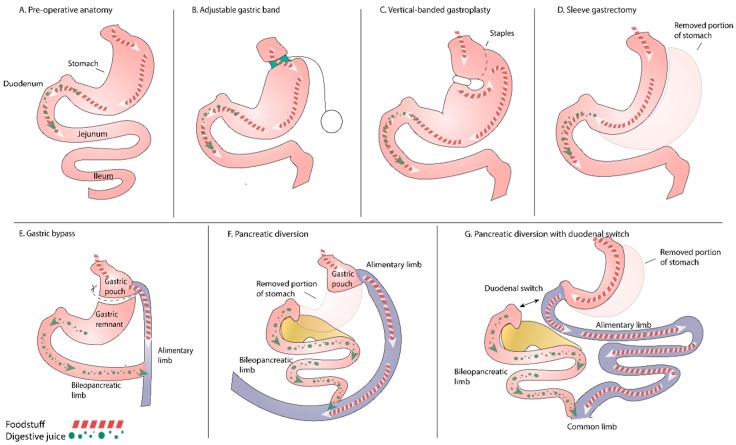
(**A**) Pre-operative anatomy; (**B**) Adjustable gastric band with subcutaneous port; (**C**) Vertical-banded gastroplasty; (**D**) Sleeve gastrectomy; (**E**) Roux-en-Y gastric bypass; (**F**) Biliopancreatic diversion; (**G**) Biliopancreatic diversion with gastric sleeve and duodenal switch.

Surgical techniques are traditionally divided into three groups (see Figure 1) [1,2]:

- food limitation operations (restrictive procedures): vertical-banded gastroplasty, adjustable gastric banding, proximal gastric bypass and sleeve gastrectomy;
- operations limiting absorption of macronutrients (limiting energy absorption): biliopancreatic diversion; and
- combined restrictive/malabsorptive operations: biliopancreatic diversion with duodenal switch or distal gastric bypass.

After bariatric surgery, the risk of possible vitamin and mineral deficiencies needs to be acknowledged [2]. All bariatric surgery procedures that bypass a portion of the small intestine might induce nutritional deficiencies. The risk of malabsorption increases proportionally with the length of bypassed proximal intestine, since this is the primary site of vitamin D, calcium, copper and iron absorption [13]. Therefore, laboratory follow-up and appropriate supplementation is necessary after all bariatric procedures [2,13,18,25]. However, despite supplementation, the prevalence of nutritional deficiencies among bariatric patients is still high, presumably due to low compliance with given recommendations in many patients [26,27,28,29,30,31].

## 3. Pancreatic Exocrine Insufficiency

Pancreatic exocrine insufficiency (PEI) refers to an insufficient secretion of pancreatic enzymes (acinar function) and/or sodium bicarbonate (ductal function) [32]. Due to the large reserve capacity of the pancreas, “mild” to “moderate” exocrine insufficiency can be compensated for, and overt steatorrhea is not expected unless the secretion of pancreatic lipase is reduced to <10% of normal levels (“severe”/“decompensated” insufficiency) [33]. Mild PEI is defined as the reduced secretion of one or more enzymes with normal bicarbonate concentration in duodenal juice and normal fecal fat excretion; moderate PEI is defined as having a reduced enzyme output and bicarbonate concentration but normal fecal fat excretion; while severe PEI has a reduced enzyme output and bicarbonate concentration plus steatorrhea [34]. Although it is commonly held that steatorrhea is the most important clinical manifestation of PEI, some studies have shown reduced absorption of fat-soluble vitamins even in patients with mild to moderate exocrine insufficiency [35,36,37]. Apart from post-surgery etiology, the most common underlying causes of PEI are acute and chronic pancreatitis, pancreatic neoplasms, celiac disease, diabetes mellitus, haemochromatosis, autoimmune diseases and HIV [32].

In the last 20 years, non-invasive methods have become the gold standards for PEI detection. While fecal fat quantification is still considered to be the gold standard, there are many disadvantages that limit its clinical applicability. (The collection of the entire stool over 3 days is required, which is unpleasant and cumbersome for patients and laboratory staff) [32]. FE1 is a very simple test for the indirect and non-invasive evaluation of pancreatic secretion. This test is widely available and only requires a small stool sample for analysis. It is widely accepted that the lower the FE1 concentration, the higher the probability of PEI. However, guidelines agree that the FE1 test is not capable of excluding mild to moderate PEI [33,34]. From a clinical perspective, it is very important that FE1 is determined in a solid stool, which significantly reduces the number of falsely low FE1 levels. FE1 levels in a watery stool could be false positives if the process of lyophilization is not performed [38]. ^13^C mixed triglyceride breath test (^13^C-MTG-BT) offers an alternative to FE1, but is not widely available [33].

The secretin-enhanced magnetic resonance cholangiopancreatography (s-MRCP) technique reveals ductal morphological alterations, simultaneously gives semi-quantitative information on functional changes, and is probably the most appropriate morphological test for the assessment of pancreatic exocrine function [39].

## 4. Exocrine Pancreatic Function after Digestive Surgery

PEI is a known and common complication after pancreatic and gastric surgery. Nakamura et al. performed a ^13^C-labeled mixed triglyceride breath test on 61 patients after pancreatoduodenectomy (PD) and found PEI in 62% of patients [40]. Gastrectomy is an important additional factor that influences the exocrine function, but also influences the digestive process in its complexicity [41]. It is known that even partial gastrectomy leads to impaired release of gastrin, pancreatic polypeptide (PP) and cholecystokinin (CCK) [42,43]. Malfertheiner et al. performed a study on rats in order to investigate adaptive changes of the exocrine pancreas occurring after distal gastric resection by different procedures (Billroth I and Billroth II) and showed that both procedures induced an organotrophic effect on the pancreas after two weeks [42]. Trophism of the exocrine pancreas in the same study was associated with an increase of amylase and trypsin, whereas lipase was not affected. It is known that afferent and efferent loop syndromes can develop following gastric surgery procedures, which might result in accelerated intestinal transit time, as well as colonization by pathogenic bacteria in the upper gastrointestinal tract with inadequate stimulation and poorly synchronized pancreatic enzyme secretion [32]. This condition is known as postcibal asynchrony and can cause PEI. As digestive products are stronger endogenous stimulators of CCK release and of pancreatic secretion than macronutrients, postcibal asynchrony is also associated with decreased endogenous stimulation [44]. However, it seems that postcibal asynchrony is just one causative factor. In addition to reducing the resorptive surface by diverting the passage of food from digestive juices via long Roux-Y- limbs, all instances of exocrine pancreatic function are affected: release, activation, degradation and effectiveness [45]. Since experimental data on animals indicated that the stomach plays an important role in the regulation of the exocrine pancreas, further studies on humans have confirmed that hypothesis [43,46]. Gullo et al. retrospectively evaluated exocrine pancreatic function in 12 patients after total gastrectomy [46]. The secretion of bicarbonate, lipase, and chymotrypsin into the duodenum in response to exogenous stimulation with secretin was significantly lower in patients after gastrectomy than in controls, and eight of the 12 patients (67%) had steatorrhea. Friess and colleagues prospectively investigated the influence of total gastrectomy on exocrine function in 15 patients, analyzing pancreatic function before and three months after surgery using the secretin-cerulein test and analysis of gastrointestinal hormone levels [43]. After total gastrectomy, patients develop severe primary PEI with decreased gastrin, decreased late postprandial PP and increased CCK levels. The authors concluded that the described hormonal changes may explain why many patients with total gastrectomy have maldigestion and weight loss postoperatively, and proposed pancreatic enzyme replacement therapy to avoid these symptoms. Armbrecht et al. evaluated the effect of peroral pancreatic enzyme replacement therapy (PERT) on abdominal symptoms, bowel habits, fecal fat excretion and oro-caecal transit time in patients after total gastrectomy for carcinoma of the stomach with Roux-en-Y anastomosis. They concluded that PERT reduces massive steatorrhea and improves stool consistency [47].

In one study including 22 patients one year after bariatric surgery (Roux-en-Y or mini-omega loop gastric bypass), exocrine pancreatic function was evaluated with fecal elastase-1 (FE1) levels. PEI was found in 9.1% of patients [48]. In addition, patients with low FE1 levels underwent magnetic resonance imaging (MRI) of the pancreas and measurement of serum nutritional markers. There were no signs of chronic pancreatitis, but at least one serological nutritional marker was below the lower limit of normal in all of the tested patients (vitamin D, vitamin A, selenium, prealbumin, zinc, copper, folic acid and iron) despite routine vitamin and mineral replacement therapy after surgery [48].

In a recently published study, 188 consecutive patients were followed for 52 months after bariatric surgery (distal and proximal Roux-en-Y gastric bypass), and exocrine pancreatic function was evaluated by clinical symptoms, FE1 and positive dechallenge-rechallenge test with pancreatic enzyme replacement therapy [45]. 31% of these patients were diagnosed with PEI.

As to the other forms of bariatic surgery, the likelihood of PEI after gastric banding (Figure 1B,C) is low since there are no anatomical changes or surgical manipulation of the gastric wall. After sleeve gastrectomy (Figure 1D), the neuronal network is impaired, and a certain degree of PEI can be expected; however, clinical studies are missing.

## 5. Conclusions

Due to the high levels of incidence after various types of bariatric surgery and the large number of bariatric surgical procedures performed, postoperative PEI might be an important clinical problem. Symptoms of PEI often overlap with sequelae of gastric bypass, making the diagnosis difficult. Steatorrhea, weight loss, maldigestion and malabsorption are pathognomonic for both clinical conditions. Altered anatomy after bypass surgery can make the diagnostic process even more difficult. Evidence suggests that the FE-1 monoclonal test is reliable for the evaluation of pancreatic function in many pancreatic and non-pancreatic disorders. It is non-invasive, less time-consuming, and unaffected by pancreatic enzyme replacement therapy. Although it cannot be considered the gold-standard method for the functional diagnosis of PEI, the advantages of the FE-1 test make it a very appropriate test for screening patients who may be at risk of this disorder [49]. Nutritional serum markers should be measured and PEI should be considered in all patients after bariatric surgery with prolonged gastrointestinal complaints and/or malabsorption. Appropriate pancreatic enzyme replacement therapy should be part of the treatment algorithm in patients with confirmed PEI or symptoms suggestive of this complication. More studies regarding the clinical implications of PEI after bariatric surgery, as well as more information about the prevalence of PEI in morbidly obese patients, is warranted.

## Figures and Tables

**Table 1:** Role of hormones on pancreatic exocrine function and food intake [4,5,7,15,16].

Hormone	Action on Pancreas Exocrine Secretion	Action on Food Intake
Cholecystokinin (CCK)	Released from enteroendocrine I cells in duodenal and ileal mucosa. Stimulates pancreatic exocrine secretion. Vagal afferent nerve fibers express several receptors for CCK.	CCK was the first gut hormone found to be implicated in appetite control. Some studies suggest that leptin and CCK may interact synergistically to induce short-term inhibition of food intake and long-term reduction of body weight.
Glucagon-like peptide-1 (GLP-1)	Released from the small intestine; inhibits hypoglycemia-stimulated exocrine secretion by direct activation of dorsal vagal complex.	GLP-1 reduces food intake, suppresses glucagon secretion and delays gastric emptying. Intravenous administration of GLP-1 is associated with a dose-dependent reduction of food intake in both normal weight and obese subjects.
Serotonin	Could activate vagal afferents to initiate enteropancreatic reflex and to stimulate pancreatic exocrine secretion. Vagal afferent nerve fibers express several receptors for serotonin.	None.
Leptin	Mainly produced and secreted by adipocytes. Controversial effect on pancreas. Could activate vagal afferents to initiate enteropancreatic reflex and stimulate pancreatic exocrine secretion. Intravenous application in rats reduces pancreatic secretion by inhibiting neurohormonal CCK-vagal-dependent mechanism. Leptin administrated in duodenum significantly stimulates pancreatic protein secretion. Significantly reduces the severity of acute pancreatitis. Vagal afferent nerve fibers express several receptors for leptin.	Regulation of food intake, energy expenditure and body weight homeostasis.
Ghrelin	Controversial effect on pancreas. Central administration in rats could activate vagal afferents to initiate enteropancreatic reflex and to stimulate pancreatic exocrine secretion. Intravenous administration in rats reduced pancreatic enzyme secretion. Vagus-dependent cholinergic pathway.	Strongly stimulates food intake. Increases adipogenesis.
Melatonin	Produced in the pineal gland and in the enteroendocrine cells of gastrointestinal mucosa and secreted into the duodenal lumen with the bile. Protects pancreas against acute damage. Dose-dependent stimulation of pancreatic exocrine secretion.	None.
Apelin	Stimulates CCK secretion. Intravenous application in rats leads to significant dose-dependent inhibition of pancreatic secretion. Intraduodenal application stimulates pancreatic secretion. Neurohormonal, CCK_1_-vagal-dependent mechanism.	Apelin is expressed in adipose tissue, suggesting adipokine functions.
Obestatin	May stimulate pancreatic protein output and trypsin activity following intravenous and intraduodenal administration (effect is dose-dependent).	Appears to have opposite actions to ghrelin on the regulation of food intake, emptying the stomach, and body weight in rodents.
Orexin-A and -B	Stimulation of pancreas exocrine secretion with orexin-A and no effect with orexin-B. Controlled by dorsal vagal complex.	Involved in the control of feeding.
